# Zebrafish disease model of human RNASET2-deficient cystic leukoencephalopathy displays abnormalities in early microglia

**DOI:** 10.1242/bio.049239

**Published:** 2020-05-07

**Authors:** Thomas Weber, Lars Schlotawa, Roland Dosch, Noémie Hamilton, Jens Kaiser, Stina Schiller, Britta Wenske, Jutta Gärtner, Marco Henneke

**Affiliations:** 1 Department of Pediatrics and Adolescent Medicine, University Medical Center Goettingen, Robert- Koch- Str. 40, 37075 Goettingen, Germany; 2 Department of Human Genetics, University Medical Center Goettingen, Justus-von-Liebig-Weg 11, 37077 Goettingen, Germany; 3 The Bateson Centre, University of Sheffield, Firth Court D31, Sheffield S10 2PT, United Kingdom; 4 Department of Haematology and Oncology, University Medical Center Goettingen, Robert- Koch- Str. 40, 37075 Goettingen, Germany

**Keywords:** Cystic leukoencephalopathy, Innate immune system, Zebrafish, Lysosomes, RNASET2

## Abstract

Human infantile-onset RNASET2-deficient cystic leukoencephalopathy is a Mendelian mimic of *in utero* cytomegalovirus brain infection with prenatally developing inflammatory brain lesions. We used an RNASET2-deficient zebrafish model to elucidate the underlying disease mechanisms. Mutant and wild-type zebrafish larvae brain development between 2 and 5 days post fertilization (dpf) was examined by confocal live imaging in fluorescent reporter lines of the major types of brain cells. In contrast to wild-type brains, RNASET2-deficient larvae displayed increased numbers of microglia with altered morphology, often containing inclusions of neurons. Furthermore, lysosomes within distinct populations of the myeloid cell lineage including microglia showed increased lysosomal staining. Neurons and oligodendrocyte precursor cells remained unaffected. This study provides a first look into the prenatal onset pathomechanisms of human RNASET2-deficient leukoencephalopathy, linking this inborn lysosomal disease to the innate immune system and other immune-related childhood encephalopathies like Aicardi-Goutières syndrome (AGS).

## Abstract (precis)

**Summary:** A zebrafish disease model of human RNASET2-deficient cystic leukoencephalopathy displays altered morphology, signs of lysosomal storage disorder, and persistent engulfment of apoptotic neurons in microglia as early disease pathology.

## INTRODUCTION

*RNASET2* is a single copy gene encoding the RNASET2 protein, the only known acidic ribonuclease in humans. Members of the RNASET2 family are found in nearly all organisms (Irie, 1999). Whereas its ribonuclease function is well understood in plants, its role in animals is still elusive (Hillwig et al., 2009). Recently, RNASET2 has been reported to degrade mammalian mitochondrial RNA in the mitochondrial intermembrane space (Liu et al., 2017). Furthermore, several studies show a critical function of human RNASET2 in the regulation of the immune system and an association with various immune-mediated diseases like inflammatory bowel disease, autoimmune thyroid disease, vitiligo and rheumatoid arthritis (Chu et al., 2011; Gonsky et al., 2017; Liu et al., 2017; Wang et al., 2015; Zhu et al., 2016).

We previously identified biallelic loss-of-function mutations in the *RNASET2* gene to cause an early-onset cystic leukoencephalopathy in humans (Henneke et al., 2009). This disorder follows an autosomal recessive inheritance and manifests as psychomotor delay, spasticity, epilepsy and normo- or microcephaly during the first year of life. Brain magnetic resonance imaging (MRI) reveals frontal and temporal lobe cystic lesions, multifocal white matter alterations and cerebral atrophy. In severe cases, clinical and neuroradiological abnormalities are already present in neonates followed by an apparently non-progressive clinical course, indicating that the active phase of the disease occurs during fetal brain development (Henneke et al., 2009, 2005). The phenotypic features of human RNASET2 deficiency are indistinguishable from the sequelae of *in utero* cytomegalovirus (CMV) brain infection. Moreover, there is a significant clinical, biochemical and neuroradiological overlap of RNASET2-deficient cystic leukoencephalopathy with Aicardi-Goutières syndrome (AGS) in some affected individuals (Tonduti et al., 2016). Remarkably, AGS is also an inherited severe neurologic condition mimicking congenital infection. AGS is caused by mutations in genes involved in nucleotide metabolism resulting in an abnormal immune response to endogenous nucleic acids (Livingston and Crow, 2016). Overall, these results suggest an involvement of the innate immune system in the disease mechanism of human RNASET2 deficiency.

Recently, we described RNASET2-deficient zebrafish representing the first animal model that mimics the human disease phenotype (Haud et al., 2011). Mutant larvae exhibited an accumulation of undigested rRNA in lysosomes within neurons by 5 days post fertilization (dpf). In adult zebrafish, we found white matter lesions on high field intensity magnetic resonance microimaging (µMRI) comparable to those observed in RNASET2-deficient infants. Moreover, we confirmed lysosomal localization of RNASET2 *in vitro*. Together, these results indicate that RNASET2-deficient cystic leukoencephalopathy is a lysosomal storage disorder, in which RNA species may be the noxious storage material provoking an inflammatory reaction similar to congenital CMV infection. This hypothesis is supported in a recently established RNASET2 rodent model. RNASET2-deficient rats exhibited altered lysosomal function and autophagy defects and most importantly, neuroinflammation in mutant brains (Sinkevicius et al., 2018).

In human RNASET2 deficiency, the main cerebral damage seems to occur during fetal brain development followed by a non-progressive neurological disease after birth. To address this hypothesis, embryogenesis of transparent zebrafish embryos provides the unique opportunity to study early central nervous system development *in vivo* (Kimmel et al., 1995). In this study, we investigated the function of cells of the innate immune system to further elucidate the pathomechanisms in *RNASET2*-mutant zebrafish embryos and early larvae. Here we show that disruption of RNASET2 leads to abnormal microglial cells with increased numbers, altered morphology and reduced lysosomal function at early developmental stages. Our results discover an unknown link between RNASET2 deficiency and the innate immune system, which also proposes a novel pathomechanism for related leukoencephalopathies like AGS.

Future studies will focus on a putatively inflammatory phenotype of RNASET2-deficient brains in larvae and juvenile fish, i.e. prior and after the maturation of the adaptive immune system.

## RESULTS

### RNASET2-deficient zebrafish larvae show altered numbers and morphology of microglial cells

To analyze early brain development in RNASET2-deficient zebrafish we used the *sa138* strain available at the Zebrafish International Resource Center (Kettleborough et al., 2013). *Sa138* fish harbor a nonsense mutation in the *RNASET2* gene that generates a premature stop codon in the second catalytic domain of the protein. The truncated gene product predictably lacks catalytic function. Homozygous *sa138* fish (mutants, *RNASET2*−/−) were fertile and born at Mendelian ratios. For subsequent experiments, we crossed this line to transgenic reporter lines that highlighted neurons, oligodendrocyte precursors (OPCs) and microglia cells as candidate cell types for early symptoms (Fig. S1). In a first experiment, we performed confocal laser scanning microscopy (CLSM) to analyze microglia in the RNASET2-deficient genetic background. Offspring from heterozygous crosses were imaged at 3 and 5 dpf covering the whole optic tectum, which is part of the dorsal midbrain. The larvae were genotyped thereafter.

**Fig. 1.**
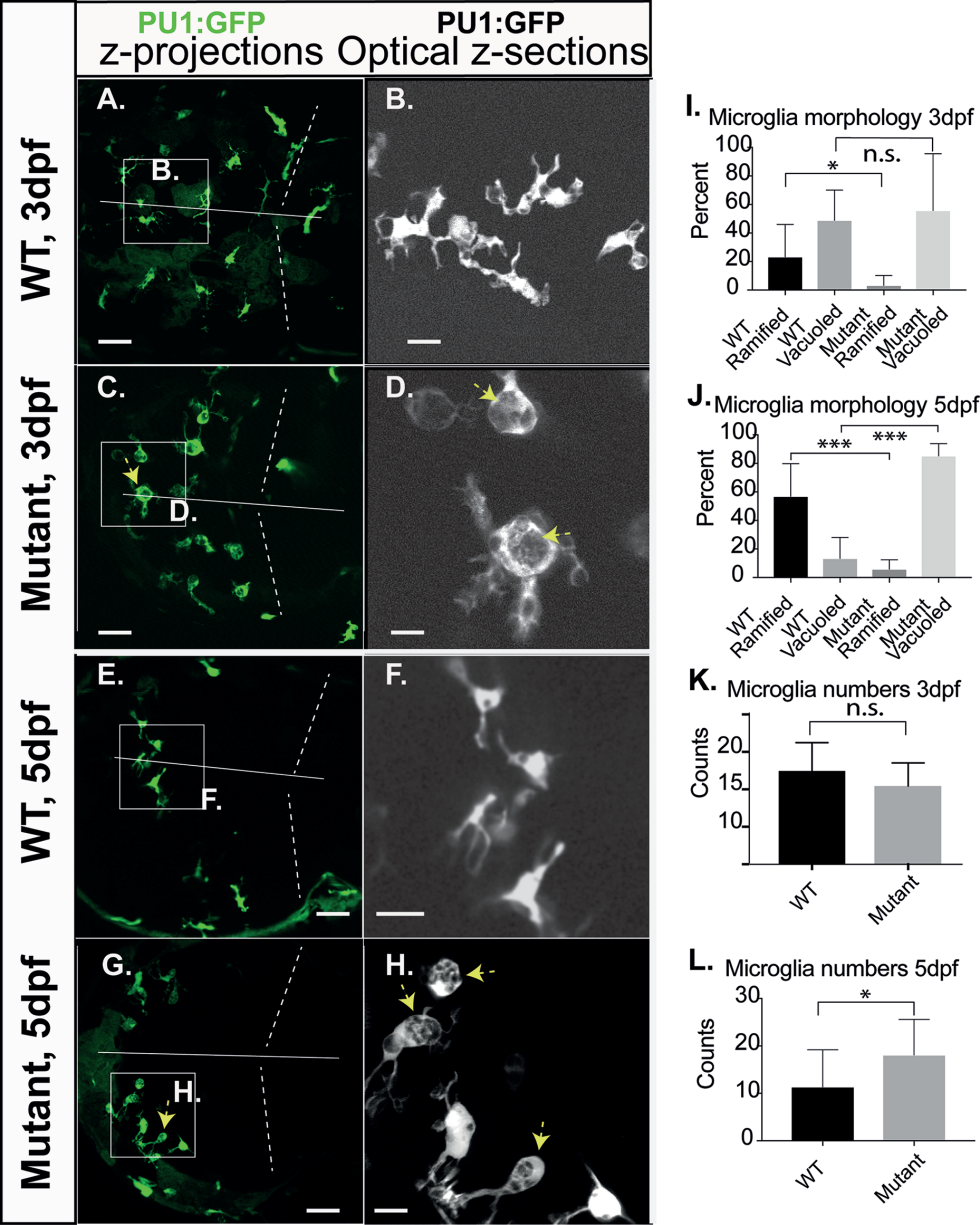
**Microglia morphology of RNASET2-deficient embryos and larvae.** Zebrafish of the transgenic reporter strain *Tg*:(PU1:GFP) were analyzed by *in vivo* CLSM. The morphology of microglia together with their total numbers was determined manually by going stepwise through the confocal z-stacks of individual larvae. These key figures were categorized, quantified and shown as averages in the respective graphs. Thus, the images shown here were selected to highlight the morphology of microglia and should not be used to deduce cell numbers or relative proportions. (A,C,E,G) Projections of confocal z-stacks through the brain showing microglia (green, arrows indicate abnormally vacuoled microglia). (B,D,F,H) Single optical sections at increased size as in the respective left panel, showing ramified and vacuoled microglia (arrows indicate abnormally vacuoled microglia). (I,J) Quantification of the morphological categories of microglia on 3 dpf (ramified mean: WT 23.2%; s.d.=22.8; *n*=14; mutants 3.2%; s.d.=7.0; *n*=9; *P*<0.5; vacuoled mean: WT 48.8%; s.d. 22.8; *n*=14; mutants 55.7%; s.d.=21.3; *n*=9; *P*>0.5) and 5 dpf (ramified mean: WT 56.9%; s.d.=23.0; *n*=12; mutants 5.8%; s.d.=6.6; *n*=14. Vacuoled mean: WT 13.3%; s.d.=14.8; *n*=12; mutants 85.2%; s.d.=8.7; *n*=14), respectively. (K,L) Averaged counts of microglia within the optical tectum on 3 dpf (mutants 10.4; s.d.=3.1, *n*=8; WT 12.5; s.d.=3.8; *n*=12; *P*>0.05) and 5 dpf (mutants 18.1; s.d.=7.9, *n*=14; WT 11.3; s.d.=8.2; *n*=12; *P*<0.05). Dotted lines, midbrain-hindbrain boundary; straight line, midline, separating left and right brain hemispheres. Scale bars: A,C,E,G: 40 µm; B,D,F,H 10 µm. *n*-numbers were generated from >7 individual litters and analyzed in seven independent CLSM-sessions.

Microglia are cells of the innate immune system and play important roles in the central nervous system (CNS) (Banati, 2003; Bilimoria and Stevens, 2015; Oosterhof et al., 2015; Sieger and Peri, 2013; Svahn et al., 2013). They quickly respond to local alterations like virus infections or acute brain lesions with rapid changes in their morphology (Mazaheri et al., 2014). We used the reporter line Tg(*PU1*:GFP) that drives expression of GFP in cells of the myeloid lineage to observe these cells in the *RNASET2*-mutant background *in vivo* (Peri and Nüsslein-Volhard, 2008; Sieger and Peri, 2013). We compared the morphology of wild-type (WT) and mutant microglia by categorizing them as ‘ramified’ or ‘vacuoled’ at 3 dpf (Fig. 1A,B, WT; C,D, mutants) and at 5 dpf (Fig. 1E,F, WT; G,H, mutants). Vacuoled microglia strikingly resembled cells predominant during normal developmental apoptosis in WT embryos containing more than five phagosomes with thin and short plasma processes (Fig. 1D,H, arrow). Microglia were described as ramified when they had more than three projections and thus appeared stellate in shape. This cellular shape is characteristic for microglia in WT brains after developmental apoptosis, i.e. at 5 dpf (Fig. 1F).

The morphology defined by those criteria was different between mutants and WT at 3 dpf. We found higher proportions of ramified cells present in WT compared to mutant embryos (Fig. 1I). In mutants, somata and vacuoles often appeared abnormally enlarged (Fig. 1D, arrows).

Highly statistically significant morphological differences between WT and mutants (*P*<0.0001) in each category became obvious at 5 dpf (Fig. 1F,H, arrows). At that time, the numbers of ramified microglia increased and became more stable in WT larvae in parallel with the cessation of developmental apoptosis of brain neurons (Svahn et al., 2013). In contrast, at 5 dpf numbers of vacuoled microglia increased in mutants (Fig. 1J). Some microglia in mutants and WT could not be classified unambiguously and were omitted. The total numbers of microglia cells were significantly increased in mutants over WT at 5 dpf, in contrast to 3 dpf (Fig. 1K,L).

The morphology of microglia is subtle at these early developmental stages as it is prone to rapid changes (Svahn et al., 2013). We therefore investigated whether the observed altered morphology of microglia (Fig. 1A–H) can be detected in siblings from independent litters of RNASET2 mutants. We therefore filtered our data to extract information about the reproducibility of this subtle phenotype. We show the morphology of mutant and WT microglia at 5 dpf from littermates of independent clutches, as well as the quantification of the relative proportions of ramified and vacuoled microglia in Fig. S2. These data demonstrate that individual larvae show different proportions of vacuoled and ramified microglia in mutants and WT as expected; however, the pattern is similar between individual clutches (Fig. S1K). Thus, we conclude that mutant microglia generally contain increased numbers of abnormally vacuoled microglia at 5 dpf in contrast to WT larvae.

Future time course experiments will help us to determine the exact time point when mutant microglia start to become abnormal, and whether they retain their abnormal morphology beyond 5 dpf.

### Microglial engulfment of apoptotic neurons persists at 5 dpf in RNASET2-deficient larvae

**Fig. 2.**
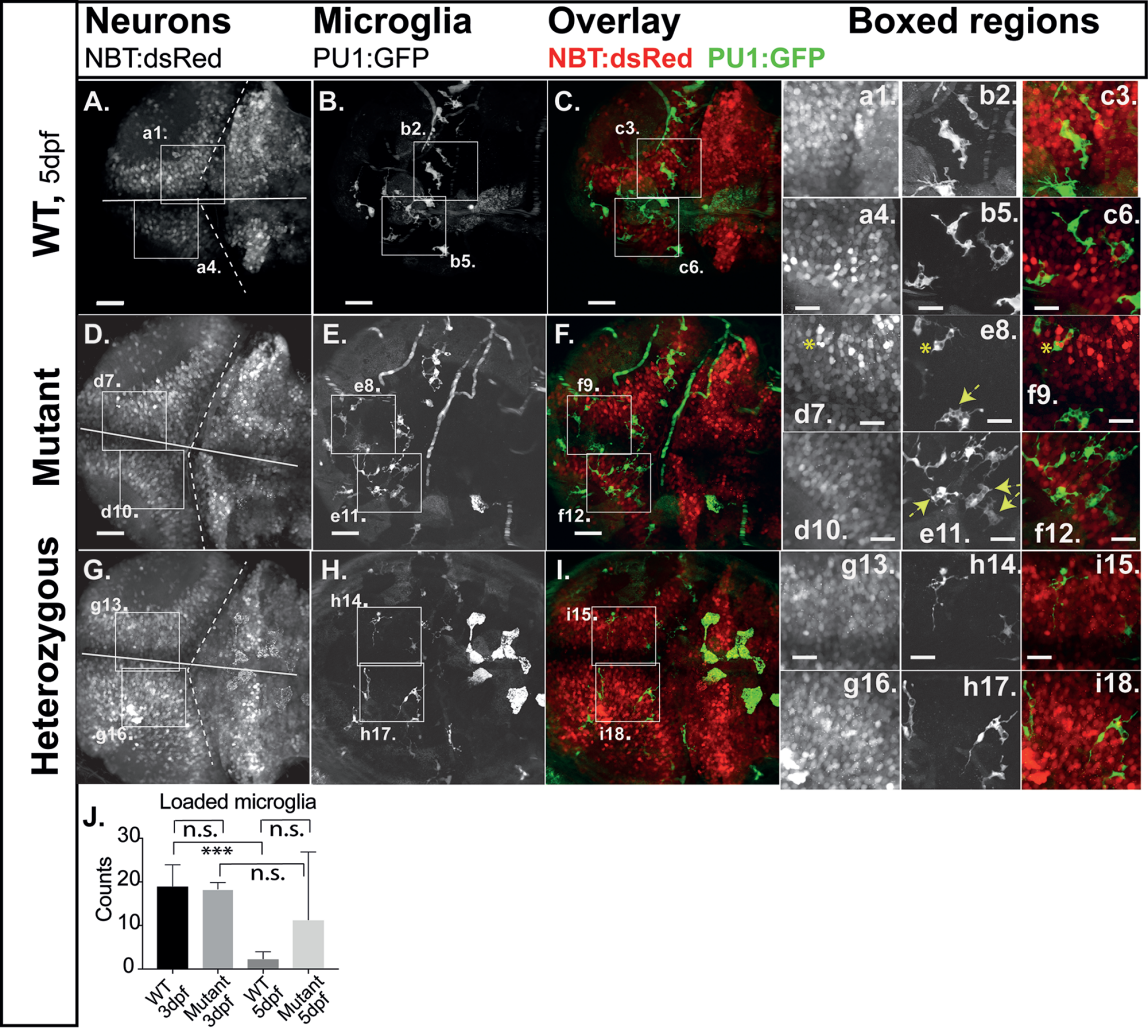
**Engulfment of apoptotic neurons.** Compound transgenic reporter strain zebrafish embryos *Tg*:(PU1:GFP)/Tg:(NBT:dsRed) were analyzed by *in vivo* CLSM at 5 dpf. Representative projections of confocal z-stacks through the brain: (A,D,G) NBT:dsRed (neurons); (B,E,H) PU1:GFP (microglia). (C,F,I) Overlays of the respective panels (A,B and C,D). Boxed regions, increased images: (a,d,g) neurons; (b,e,h) microglia; (c,f,i) microglia with engulfed apoptotic bodies of neurons (loaded microglia, asterisks; vacuoled microglia, arrows). (J) Quantification of loaded microglia (3 dpf, see Fig. S3: WT 19.0; *n*=5; mutants 18.3; *n*=3; *P*>0.5; 5 dpf: WT 2.3: s.d.=1.8; *n*=6; mutants 11.3; s.d.=15.6; *n*=7; *P*>0.5). Scale bars: 40 µm. E,F show expression of GFP in circulating cells as observed in about 20% of PU1:GFP-positive larvae. *n*-numbers were generated from one to three individual litters and analyzed in one to three independent CLSM-sessions.

A key function of microglia is the phagocytic removal of neurons and other cells during developmental apoptosis in the zebrafish brain, which predominantly happens between 20 and 74 hours post fertilization (hpf) (Cole and Ross, 2001). In the tectum and cerebellum such apoptosis declines after 3 dpf (Cole and Ross, 2001). To highlight neurons *in vivo* we used the pan-neuronal reporter line Tg(*NBT*:dsRed), (Peri and Nüsslein-Volhard, 2008; Sieger et al., 2012) in which cytoplasmic DsRed-based fluorescence is driven by elements of the neuron-specific *beta*-tubulin (*NBT*) promoter from *Xenopus*. Engulfment of almost all apoptotic neurons by microglia has been reported to happen during normal developmental apoptosis (Mazaheri et al., 2014; Peri and Nüsslein-Volhard, 2008; Svahn et al., 2013). Hence, in the compound transgenic reporter line Tg(*NBT*:dsRed/*PU1:*GFP), green fluorescent microglia cells that contain red fluorescent particles (loaded microglia) highlight apoptotic events *in vivo* (Fig. 2; Fig. S3, boxed regions d7,e8,f9 asterisk). As expected, on 3 dpf microglia with inclusions of red fluorescent neurons were most commonly observed in WT embryos (91.6%) and only few microglia without inclusions, reflecting the correlation between neuronal apoptosis and microglia function (Fig. 2D–F and corresponding boxes). We found no significant difference between WT and mutant on 3 dpf (Fig. S3). By 5 dpf only a few WT brains contained microglia with inclusions of red fluorescent neurons (12.8%) and numbers were significantly reduced in comparison to 3 dpf WT fish. Heterozygous larvae resembled WT larvae (Fig. 2G–I). This reflects the normal cessation of developmental apoptosis and indicates efficient clearance of apoptotic bodies by microglia. Interestingly in mutants, we still found microglia with inclusions of red fluorescent neurons in 32.7% of analyzed brains at 5 dpf. Therefore, the highly significant decline between 3 dpf and 5 dpf that was detected in WT could not be observed in mutants; the differences between 3 dpf and 5 dpf in mutants were trending albeit not statistically significant in a two-tailed unpaired *t*-test (loaded microglia, Fig. 2J).

Although similar amounts of larvae were tested in WT and mutants, we think that this could be explained with the sample sizes being too low.

We speculated that the presence of microglia with inclusions of red fluorescent neurons and an increased number of microglial cells in mutants on 5 dpf could result from abnormal entry of neurons into apoptosis after 3 dpf, or from delayed clearance of apoptotic neurons in mutant microglia.

### Neurons and Olig2-positive cells in the hindbrain develop normally until 5 dpf

**Fig. 3.**
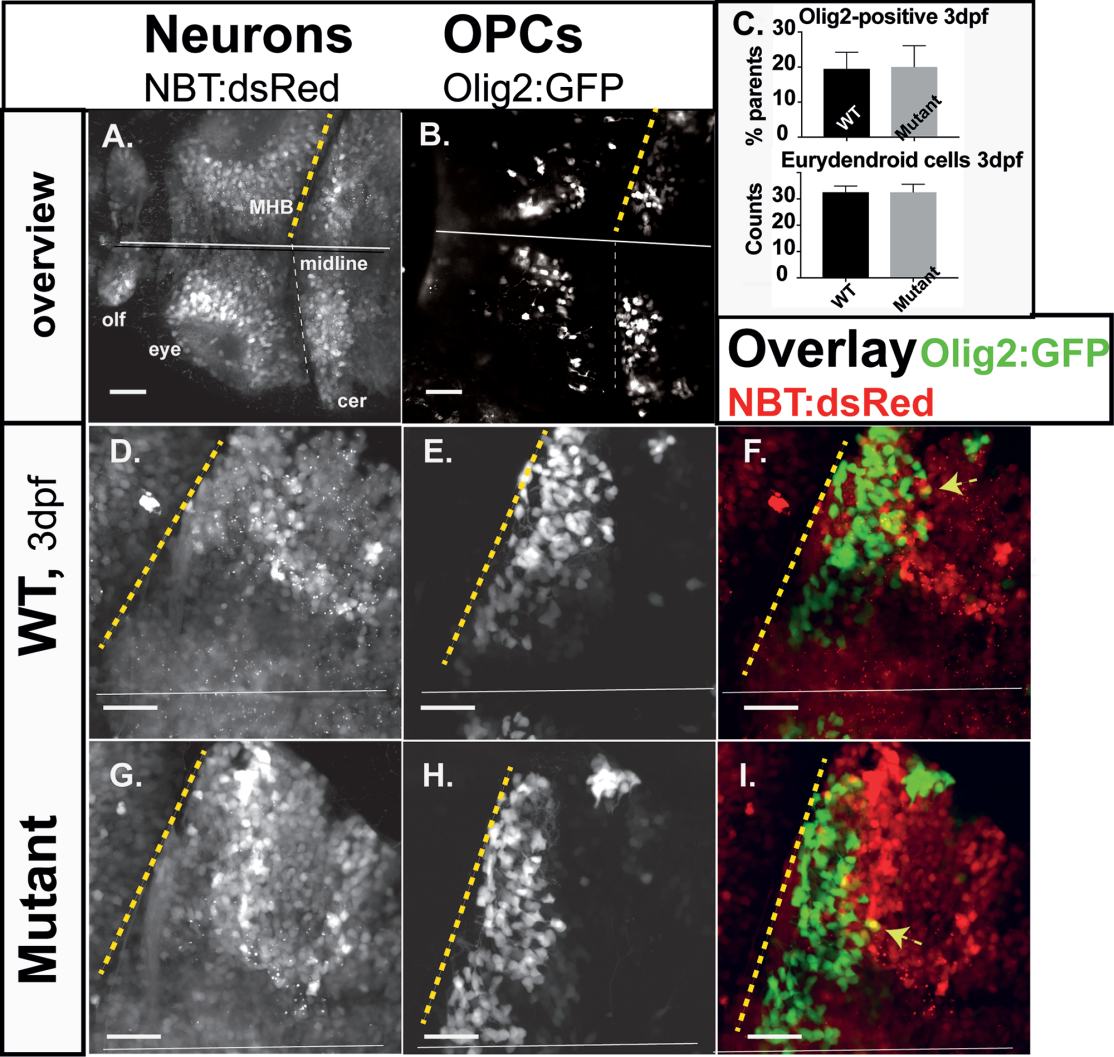
**Neurons and oligodendrocytes in the developing hindbrain.** Zebrafish larvae of compound transgenics *Tg*:(NBT:dsRed)/*Tg*:(Olig2:GFP) analyzed by *in vivo* CLSM. Projections of confocal z-stacks highlight neurons and OPCs at 3 dpf. (A,B) Overview; brain of a WT larvae, showing neurons (NBT:dsRed) and OPCs (Olig2:GFP). (C) Relative proportions of OPCs (average OPCs: WT 19.53%; *n*=7; mutants 20.8%; *n*=4; *P*>0.5) and counts of developing eurydendroid cells (average ECs: mutants 32.6; *n*=3; WT 32.6; *n*=3; *P*>0.5) in the right cerebellar hemispheres. Expression of both fluorophores in F and I is indicated by yellow arrows. Projections of representative confocal z-stacks through the cerebellum on 3 dpf (D,E,G,H). Scale bars: 40 µm. *n*-numbers were generated from one litter and analyzed in one CLSM-session.

White matter is mainly composed of myelinated axons and oligodendrocytes, which create the myelin sheath, while grey matter mainly consists of neuronal cells. On brain imaging, infants with RNASET2 deficiency predominantly reveal a leukoencephalopathy with multifocal white matter alterations, including calcifications in supratentorial but also cerebellar grey and white matter regions (Henneke et al., 2009, 2005; Tonduti et al., 2016). To highlight OPCs *in vivo* we used the GFP-reporter strain Tg(*Olig2*:GFP) in the RNASET2-mutant background for CLSM (Park et al., 2007). We also made use of the pan-neuronal reporter line Tg(*NBT*:dsRed) (Peri and Nüsslein-Volhard, 2008). Mutant *NBT*-DsRed and *Olig2*-GFP-expressing cells in the cerebellum appeared normal at 3 dpf (Fig. 3A,B,D–I). Their relative proportions were determined by flow cytometry, which revealed no significant differences between WT and mutants (Fig. 3C). Simultaneous expression of DsRed and GFP in compound transgenics [Tg(*NBT*:dsRed/*Olig2*:GFP)] highlight maturating eurydendroid cells (ECs), a distinct neuronal population within the developing cerebellum, receiving input from parallel fibers and precursor cells (Bae et al., 2009) (Fig. 3F,I, arrows). Their numbers were also unaltered in mutants, based on manual counts through the future corpus cerebellum within composite z-stacks at 3 dpf (Fig. 3C). Taken together neurons and OPCs in the developing brain of RNASET2-deficient larvae did not show any abnormalities in numbers or morphology.

### No indication for irregular cell death in RNASET2-deficient larvae at 5 dpf

To detect apoptosis or a different mode of cell death in WT and mutant larvae we used Annexin V (AV)/Propidium Iodide (PI) cell staining following a modified protocol that involves dissociation of the larvae and quantification by flow cytometry (Sakowski et al., 2012). Notably, this approach allows precise comparisons of staining intensities between WT and mutants with AV (apoptosis) or AV and PI (necrosis), revealing the proportions of necrotic and apoptotic cells within the total population. To validate our assay we used WT samples from embryos on 2 dpf that showed increased amounts of AV-stained cells as compared to WT larvae on 5 dpf. This reflects the dynamics of normal developmental apoptosis in zebrafish brains (Fig. S4A) rendering the assay a suitable tool for our analysis.

Next, we compared the proportions of apoptotic and necrotic cells in WT and mutant embryos at 5 dpf, the time with obvious differences in microglial numbers and morphology. Neither AV nor AV/PI double positive cells were significantly different (Fig. S4B).

To refine our analysis we used the same dissociation and flow cytometry protocol and determined relative proportions of neurons and OPCs using fluorescent compound reporter lines [Tg(*NBT*:dsRed) and Tg(*Olig2*:GFP), see below] in an RNASET2-deficient background. Proportions (shown as percentage within the total number of cells) were not significantly different between mutant and WT larvae on 5 dpf (Fig. S4C). However, average endogenous fluorescence emission in the red spectrum from neuronal *NBT*:dsRed was increased in mutants likely reflecting additional red fluorescence from neurons as inclusions in microglia (Fig. S4D). In conclusion, the increased number of microglia is not caused by enhanced cell death in RNASET2-deficient larvae at 5 dpf.

### Increased lysosomal staining in microglia from RNASET2-deficient larvae

RNASET2 is the only acidic ribonuclease in humans suggesting a key role for RNASET2 in lysosomes (Haud et al., 2011). We previously observed *in vitro* that depletion of RNASET2 leads to an accumulation of lysosomes in EK cells, probably as a compensatory mechanism (Haud et al., 2011). Lysosomes are especially important in cells with active phagocytosis such as microglia. We therefore hypothesized that the lower rate of digested phagocytosed apoptotic bodies in mutant microglia originates from a lysosomal dysfunction. We used LysoTracker Red staining of single cells that had been obtained by dissociating larvae with collagenase, and quantification by flow cytometry.

The rationale for this approach rather than using, e.g. CLSM and manual counting from LysoTracker Red-stained larvae, was that isolated cells allow for highly reproducible staining and solid quantification. A disadvantage of this approach is that we are missing microglia-specific data as could be obtained with CLSM-analyses.

**Fig. 4.**
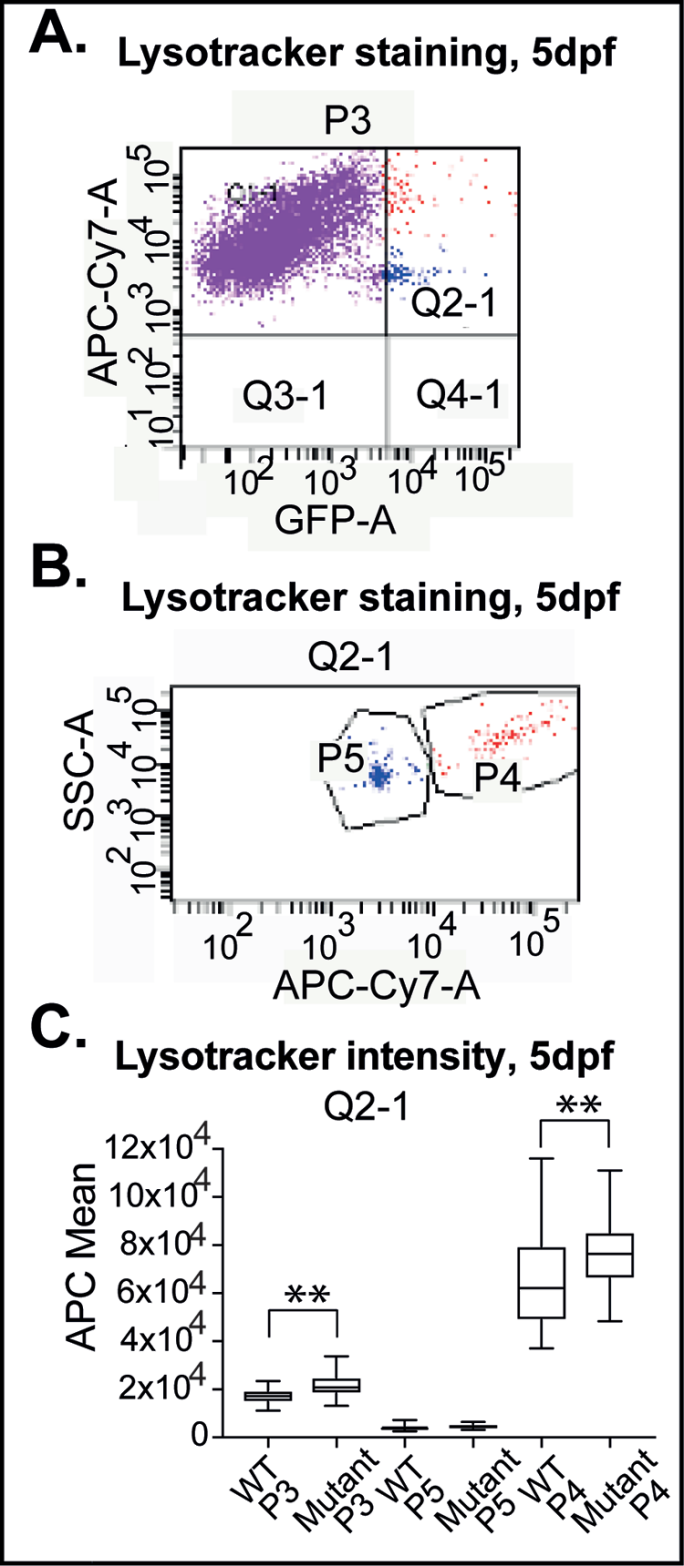
**Lysosomal staining of dissociated** **cells from** **WT and mutant larvae.** Larvae analyzed by flow cytometry after single cell dissociation and staining. (A) Scatter plot of whole cell population PU1:GFP (GFP-A)/LysoTracker Red (APC-Cy7-A) (P3), gating LysoTracker Red-stained cells (quadrants Q1-1, Q2-1) and GFP-positive cells (quadrant Q2-1). (B) Quadrant Q2-1, containing GFP- and LysoTracker Red-positive cells, as SSC-A plot, reveals two distinct cell populations P5 and P4. P4 are heterogeneous in size and granularity, P5 are homogenous in size, reduced granularity (APC mean P4: 78,925, minimum 37,075 maximum 116,000; P5: 4,635, minimum 2,579, maximum 7,214). (C) Quantification of average LysoTracker Red intensities (APC mean) of all three populations (P3: WT 16,913.4; *n*=22; mutants 21,360.2; *n*=25; *P*<0.01, P4: WT 64,873.9; *n*=22; mutants 77,090.6; *n*=25; *P*<0.01, P5: WT 4171; mutants 4695; *P*>0.05) in WT and RNASET2-deficient larvae, respectively. *n*-numbers were generated from nine litters and analyzed in four independent sessions.

First, LysoTracker Red staining (APC-Cy7-A) was measured within the whole cell population (P3) on 5 dpf (Fig. 4,C). Cells from mutant larvae took up 26.29% more LysoTracker Red than WT larvae, consistent with lysosomal dysfunction (Fig. 4C).

Since the transcription factor PU1 is expressed in myeloid cells – a mixed population – embryos and early postembryonic larvae express GFP not only in microglia but also in cell populations outside the brain parenchyma, including circulating blood cells (Fig. 2E,F) (Hsu et al., 2004). Plotting GFP-A against APC-Cy7-A revealed two distinct subpopulations (GFP-A versus APC-Cy7-A) in flow analyses, detectable in all WT and mutant samples, denoted as P4 and P5 (Fig. 4A,B, P3 quadrant Q2-1). On 5 dpf emission differed by more than 15-fold between these two populations (APC mean: P4 64.874; P5 4.171). Increased side scatter (SSC-A) showed that cells in P4 were heterogeneous in size and granularity, while P5 cells had lower granularity and were very homogenous, and thus formed a dense cloud of cells in scatter plots. Note the large range of staining intensities within P4 versus P5 (Fig. 4B). Based on our side- and forward scatter data we suggest that the two populations P4 and P5 are circulating cells (Fig. 2E,F) and monocytes, respectively. However, a substantial validation would require more sophisticated cytologic analyses, including cell sorting, which are beyond the scope of this study.

When we compared WT and RNASET2 mutants, we found that cells within the P4 population of mutants take up 18.8% more LysoTracker Red stain on 5 dpf than those of WT larvae. In contrast, differences were not significant between WT and mutants within the P5 population (Fig. 4C). Thus, we conclude that RNASET2-deficient phagocytic cells display an increased acidic compartment because of lysosomal dysfunction.

## DISCUSSION

We studied potential pathophysiological disease mechanisms of an early onset childhood leukoencephalopathy caused by loss-of-function mutations in the gene encoding the ribonuclease RNASET2. Using a zebrafish disease model, we analyzed early developmental stages by confocal live imaging in genetic contexts of fluorescent reporter lines for OPCs, early neurons and microglia cells – all candidate cell types for the emergence of early symptoms. Our imaging data were complemented by live cell staining and flow cytometry-based quantitative analyses.

Microglia are the major cell type of the myeloid lineage in the CNS. We found evidence for microglia abnormalities as to morphology, numbers and presumptive lysosomal function in the developing brain of RNASET2-deficient larvae. These abnormalities were already detectable in embryos on 3 dpf and became evident in larvae on 5 dpf. At that time physiological developmental apoptosis of neurons ends and is followed by the beginning of myelination of axons by oligodendrocytes in the CNS of zebrafish larvae between 4 dpf and 7 dpf (Brösamle and Halpern, 2002). OPCs were of different interest in our study, since they develop into oligodendrocytes, the myelinating cells of the CNS that form a major part of white matter, which is mainly affected in patients with RNASET2-deficient leukoencephalopathy and a hallmark of the disease. However, OPCs remained unaffected in our zebrafish disease model at early timepoints. We conclude that the morphological phenotype of RNASET2-deficient larvae at 5 dpf is weak and in the brain appears to affect only microglia. Overall brain morphology looked inconspicuous, also as generally seen by analysis of the scattered light during confocal microscopy. We observed subtle phenotypes such as the delicate morphology of microglia and their yet unsettled phagocytic behavior as well as the robust lysosomal abnormalities, a more general phenotype.

Thus, our study could show for the first time that in the early stages of CNS development brain abnormalities already occur in the population of microglial cells.

### Is there developmental delay in RNASET2-deficient embryos?

The morphological differences in microglia such as the presence of vacuoled microglia, a trend towards increased amounts of undigested neuronal material in microglia, and increased lysosomal compartments in distinct populations within the myeloid lineage could be an indicator of delayed brain development. However, we detected no evidence for abnormalities in cells other than microglia in the maturating zebrafish on 5 dpf, and the developing brains of mutants and WT were similar. Therefore, delayed brain development in the absence of RNASET2 gene function is not a very likely explanation for these differences.

### RNASET2-deficient leukoencephalopathy exhibits signs of a microglial lysosomal storage disorder

About 50 lysosomal storage disorders have been described in humans, categorized according to the specific biologic material that is excreted or accumulates within lysosomes. Consequently, specific cell types or organs are more affected than others (Ballabio and Gieselmann, 2009). From our previous study on zebrafish we concluded that RNASET2-deficient cystic leukoencephalopathy could be a lysosomal storage disorder, in which rRNA species are the best candidate for the noxious storage material (Haud et al., 2011). In human lysosomal storage disorders with neurodegenerative course such as mucopolysaccharidosis (MPSI, MPSIII), a role of microglia in the disease mechanism is well characterized including the analyses of mouse models for the diseases (Ohmi et al., 2003). In MPS, the morphology of microglia resembles that observed in our study. Microglia are activated and trigger a fatal inflammatory response. It is believed that the ensuing neuronal apoptosis is a cumulative secondary effect, in addition to neuronal stress and cell damage due to lysosomal overload in microglia as the primary defect. In the human microgliopathy, the hereditary diffuse leukoencephalopathy with axonal spheroids (HDLS) patients carry a mutation in the microglia specific colony stimulating factor receptor 1 (CSF1R) gene (Rademakers et al., 2012; Rietkotter et al., 2015). The disease, which also involves the CNS white matter, primarily arises from microglial dysfunction. This leads to a secondary disruption of axonal integrity and finally to a progressive axonal demyelination. The disease onset in MPS and HDLS occurs after apparently normal brain development and in both cases microglia play a role as secondary trigger.

Abnormal microglia seen in our study resemble phagocytosing microglia during developmental apoptosis in the embryonic zebrafish brain. Cells already showed an abnormal morphology and displayed inclusions of undigested neurons at 3 dpf and 5 dpf. We found evidence for signs of a lysosomal storage disorder. Defects in lysosomal function that likely affect downstream cellular functions like autophagy, which result in more complex microglia dysfunction beyond pure ribonuclease failure, are yet to be elucidated. Interestingly, a recent manuscript reports microglia abnormalities in a CRISPR/Cas9 generated RNASET2-deficient zebrafish line with increased numbers of TUNEL-positive cells at 5 dpf, indicative of higher levels of apoptosis (Hamilton et al., 2020). This observation could serve as an alternative/additional explanation for increased numbers of microglia at 5 dpf that we also found in our study. However, we did not detect an increase of apoptosis by a different method, AV/PI staining and flow cytometry. Further studies will be required to elucidate differences between the CRISPR/Cas9 RNASET2-deficient zebrafish line and the sa138 line used in our experiments. Nonetheless, the same conclusion that microglia display impaired clearance activity adds further evidence to the involvement of the innate immune system at early stages of the disease.

Clearly, mutant zebrafish larvae at 5 dpf highlight an early critical time window in the pathogenesis of RNASET2 deficiency, right after developmental apoptosis in the CNS and before the myelination of axons and the development of a characteristic disease phenotype. Abnormal microglia might trigger an inflammatory reaction as already described in MPS and other neurodegenerative disorders with involvement of the immune system. Such an inflammation could coincide with myelination of axons or appear later, thereby causing lesions in the white matter of the brain, as seen in mutant adult fish and patients. Our results indicate that white matter abnormalities in patients are rather related to intrauterine dysfunction of microglia in brain development than to a primary defect of oligodendrocytes. However, an overlaying delay in developmental apoptosis cannot be ruled out based on our data set. This is in line with the observation that in infants carrying loss-of-function mutations in RNASET2 the active phase of the disease and the main cerebral damage occur during prenatal stages followed by a rather static disease course postnatally. Further studies will have to provide in-depth analysis of the involvement of the immune system in early developmental stages of RNASET2-deficient zebrafish, a major focus addressed by our future studies.

### Non-cell autonomous roles of RNASET2 in the brain

Human RNASET2 has been described as a tumor-antagonizing gene and as a putative stress-sensor (Acquati et al., 2011; Lualdi et al., 2015). Moreover, cell autonomous and non-cell autonomous roles of this secreted enzyme have been revealed in several cancer models. In zebrafish, the loss of RNASET2 gene function induces a lysosomal storage disorder in distinct populations of myeloid cells, probably by a cell autonomous mechanism, as we show here. It would be interesting to investigate whether non-cell autonomous mechanisms are involved the RNASET2-deficient brain, e.g. by performing cell specific rescue experiments.

### Similarities and dissimilarities with other leukoencephalopathies

In RNASET2-deficient patients, the full clinical phenotype is already present within the first year of life (Henneke et al., 2005). No progressive clinical deterioration occurs, i.e. the further course of the disease is static. This is different from other immune-related inherited childhood leukoencephalopathies such AGS. In AGS, the classical clinical course is characterized by an active period of neurological regression in the first months of life followed by a stable further course, leaving the child with profound disabilities after an apparently normal initial development (Livingston and Crow, 2016). However, these disorders do not only show a significant clinical and neuroradiological, but also an pathophysiological overlap since both have an impact on nucleotide metabolism and/or sensing, which leads to an abnormal immune response (Crow et al., 2006; Reijns et al., 2012; Rice et al., 2009; Sundal et al., 2015). While a strong neuroinflammatory component is present in AGS patients, as well as in RNASET2-deficient rats (Sinkevicius et al., 2018), such a feature has not been clearly verified yet in patients with RNASET2 deficiency, probably due to the low number of identified patients. The presence of abnormal microglial cells in early mutant larvae in our study provides first experimental evidence that early symptoms in RNASET2-deficient newborns are linked to the innate immune system. Thus, RNASET2 deficiency and AGS might be more closely related than previously assumed (Tonduti et al., 2016). The different onsets of signs and symptoms in RNASET2 versus AGS patients may be related to the affected protein and its function at different time points during ontogeny as well as to the particular nucleotide species being involved. Based on our data, the catalytic activity of RNASET2 is crucial for early microglial function. In contrast, the proteins defective in AGS may become more important at later neurodevelopmental stages.

## MATERIALS AND METHODS

### Animal husbandry and screening

Zebrafish were maintained under 14 h light/10 h dark cycles at 28°C in groups of 10–30 adults according to legal regulations (EU-Directive 201_63). We used standard procedures to cross zebrafish for egg production and to prepare them for experimental analysis (Weber, et al., 2016). Using a fluorescence stereomicroscope (Zeiss, Germany), embryos expressing the expected fluorescence patterns were selected and kept in separate dishes. Usually we maintained up to 20 embryos from one to three different clutches for an experimental session. In all sessions we used sibling controls.

### Genotyping

Zebrafish were genotyped by extracting genomic DNA from tail biopsies of adult fish (3–4 months) or from whole embryos using standard procedures (Haud et al., 2011). Genomic DNA-sequencing was performed after preamplification and cleanup of an approximately 400 bp fragment containing the region of interest using the Sanger sequencing method. Primers for sequencing were: T2GenotF1: TGTCTTAGGCATTGTCGGTTTC (preamplification); T2GenotR1: CTTGTGGTAGAGTTCGAGAGC (preamplification and sequencing). Sequences were processed using the programs ApE v2.0.47 and SnapGene Viewer v4.1.9. Fish were genotyped after the respective analyses. Thus, experiments were performed ‘blind’.

### CLSM

Embryos were anesthetized with 0.04 mg/ml tricaine (MS-222, Sigma-Aldrich), embedded in 1% low melting agarose, positioned and mounted on custom-made imaging chambers. Microscopic analysis was performed using an inverted laser scanning confocal microscope (Olympus) or a LSM780 (Zeiss) and a 40× Apochromat water immersion objective (NA 1,2; Zeiss). Confocal z-stacks through the brain imaged from fluorescent reporter lines of approximately 512 nm thickness per image were taken. Images were processed using Adobe photoshop/illustrator CS5.1, ImageJ 1.48b and Imaris 7.1.0. The adjustment of contrast levels and Gaussian blurring from the raw data – if applicable – was performed exclusively using whole images.

### Embryo dissection

Embryos were treated with collagenase II in Ringers' solution (0.4 U/ml) at 28°C and shaking at 700 rpm for 30–45 min with three manual trituration steps using a 100 µl pipette tip. Dissociated cells were then centrifuged for 3 min at 5000 rpm and re-dissolved in 200 µl PBS, passed through a 100 µm cell strainer, stained and immediately analyzed by flow cytometry.

### Live cell staining

Single cells from dissected larvae were stained with 1 picomolar LysoTracker^R^ Deep Red (Molecular Probes) for 15 min, and analyzed by flow cytometry as described below.

### Cell death assays

After dissection of larvae, cell pellets were dissolved in 150 µl AV binding buffer (BioLegend) in FACS tubes. Cells were stained for 15 min in the dark by adding 5 µl APC/AV conjugate (BioLegend; Koopman et al., 1994) and 10 µl propidium iodide from a 50 µg/ml stock solution following flow cytometry using the APC-Cy7-A (633 nm laser line) and PE-A filter sets. Gates were defined for each set of experiments by using respective unstained controls. The averaged percentages of  WT and mutant samples in different quadrants, defining apoptotic and necrotic cells, respectively, were compared.

### Flow cytometry

Cells were analyzed using a FACS Canto II flow cytometer equipped with FACSDiva software (BD Biosciences). Between 1–3×10^4^-events from each dissected embryo were analyzed within the Hoechst 33342-positive population to get statistically significant results and eliminate potential signals from cell fragments.

For detecting neuron-based DsRed, PE-A filter settings were used, and GFP-A filter sets for oligodendrocytes and microglia. LysoTracker Red-stained cells were measured using the APC-Cy7-A filter set after excitation at 633 nm.

### Cell counting

Cells were counted from confocal z-stacks of about 100 µm thickness. These had been obtained by CLSM during live imaging session using fluorescence reporters for the respective cell type, by scanning from the dorsal top through the optic tectum in z-direction thereby covering the optic tectum in x–y direction. Thereby confocal optical slices of 500–1000 nm thickness were generated. Cell counting was performed manually by going stepwise through the optical sections in z-direction.

### Statistical analyses

Graphpad Prism 7 software was used to evaluate the significances of differences between mutant and WT parameters in unpaired, two-tailed Student's *t*-tests or *F*-tests for variance, where *P*-values of <0.05 were considered significant (*), <0.01 (**) and <0.001 (***) as highly significant; *P*>0.05 was considered not significant. *n*-numbers represent the numbers of analyzed animals.

Thus, *n*-numbers reflect the numbers of biological replicates and are indicated in the figure legends. See also the section Flow Cytometry. To obtain the indicated *n*-numbers, experiments had to be performed consecutively, in individual sessions, using up to 20 embryos or larvae in each session. The numbers of sessions are indicated in the respective figure legends.

## Supplementary Material

Supplementary information
