# Supplementary material for: Zebrafish disease model of human RNASET2-deficient cystic leukoencephalopathy displays abnormalities in early microglia
Source: Biol Open. 2020 May 7;9(5):bio049239. doi: 10.1242/bio.049239 (PMC7225086; doi:10.1242/bio.049239)
Supplement: Supplementary information [file biolopen-9-049239-s1.pdf]

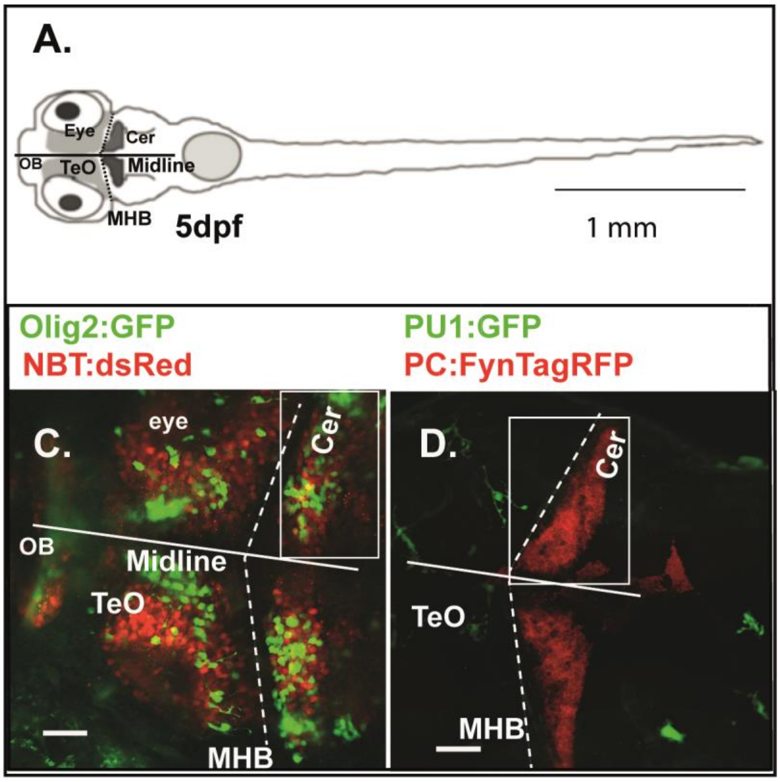

Figure S1

Transgenic zebrafish lines and analyzed zebrafish brain regions

A: Cartoon of a zebrafish larvae on 5 dpf showing the regions analyzed in this study. TeO Tectum opticum; OB Olfactory bulb; Cer Cerebellum; MHB Midbrain-hindbrain boundary. **B:** WT zebrafish larvae of compound transgenics *Tg*:(NBT:dsRed)/*Tg*:(Olig2:GFP) analyzed by *in vivo* CLSM. Projection of a confocal z-stack highlighting neurons (red) and oligodendrocyte precursor cells (green) on 5 dpf. **C:** WT zebrafish larvae of compound transgenics *Tg*:(PU1:GFP)/ *Tg*:(PC:FynTagRFP) analyzed by *in vivo* CLSM. Projection of a confocal z-stack highlighting microglia (green) and purkinje cells (red) on 5 dpf. Size markers 40 µM.

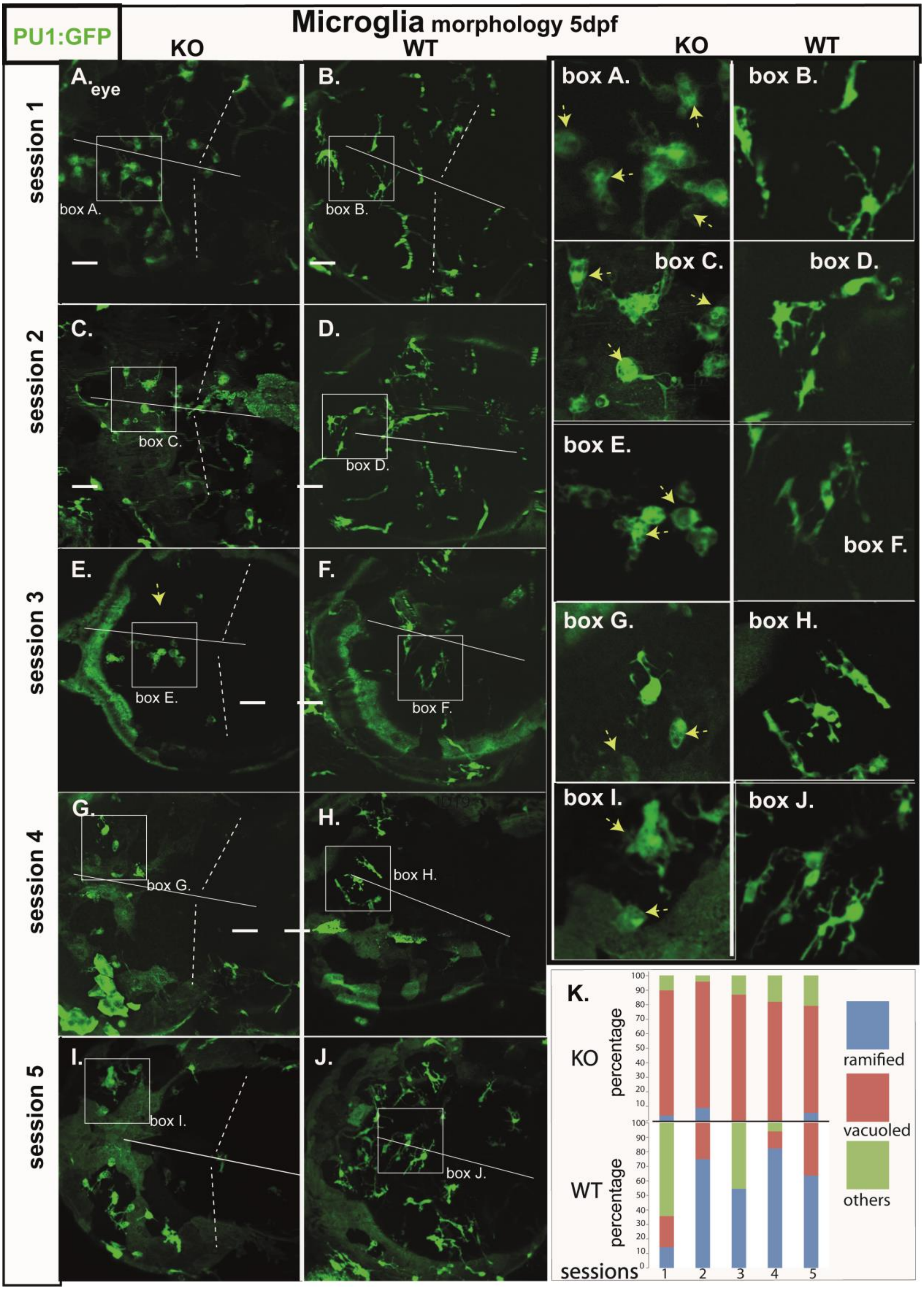

Biology Open • Supplementary information

Figure S2

Microglia morphology of RNASET2-deficient embryos and larvae in independent clutches.

Zebrafish of the transgenic reporter strain *Tg*:(PU1:GFP) were analyzed by *in vivo* confocal laser scanning microscopy (CLSM). Data shown in Figure 1 were filtered to show the morphology of mutant and WT larvae from 5 independent clutches (session 1-5).

A-J Projections of confocal z-stacks through the brain showing microglia (green, arrows). **Box A.-J** (right panels) showing ramified and vacuoled microglia at increased size as in the respective images **A.-J.** (left panels). Mutant and WT larvae from the same session are opposed (**Session 1**: **A.** mutant & **B.** WT**; Session 2**: **C.** mutant & **D.** WT; **Session 3**: **E.** mutant *&* **F.** WT; **Session 4**: **G.** mutant & **H.** WT; **Session 5**: **I.** mutant & **J.** WT

K: Depiction of the morphological categories (ramified, vacuoled, others) of microglia at 5 dpf from 5 sessions.

Biology Open • Supplementary information

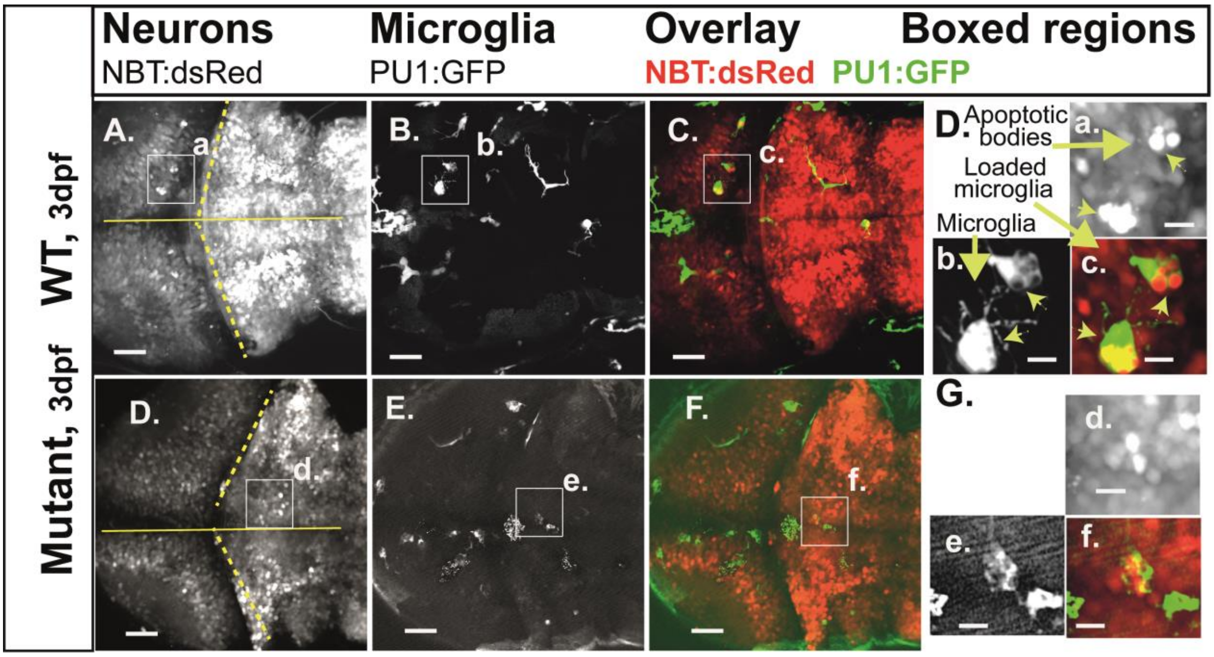

Figure S3

Images of engulfed apoptotic neurons at 3 dpf

Compound transgenic reporter strain zebrafish embryos *Tg*:(PU1:GFP)/*Tg*:(NBT:dsRed) were analyzed by *in vivo* CLSM at 3 dpf. Representative projections of confocal z-stacks through the brain: **A, D**: NBT:dsRed (neurons); **B ,E**: PU1:GFP (microglia). **C, F:** overlays of the respective panels (A,B and C,D). Boxed regions, increased images: **a., d.**: apoptotic neurons; **b., e.**: microglia; **c. f.**: microglia with engulfed apoptotic bodies of neurons (= loaded microglia; arrows). Size markers 40 and 10 µm.

Biology Open • Supplementary information

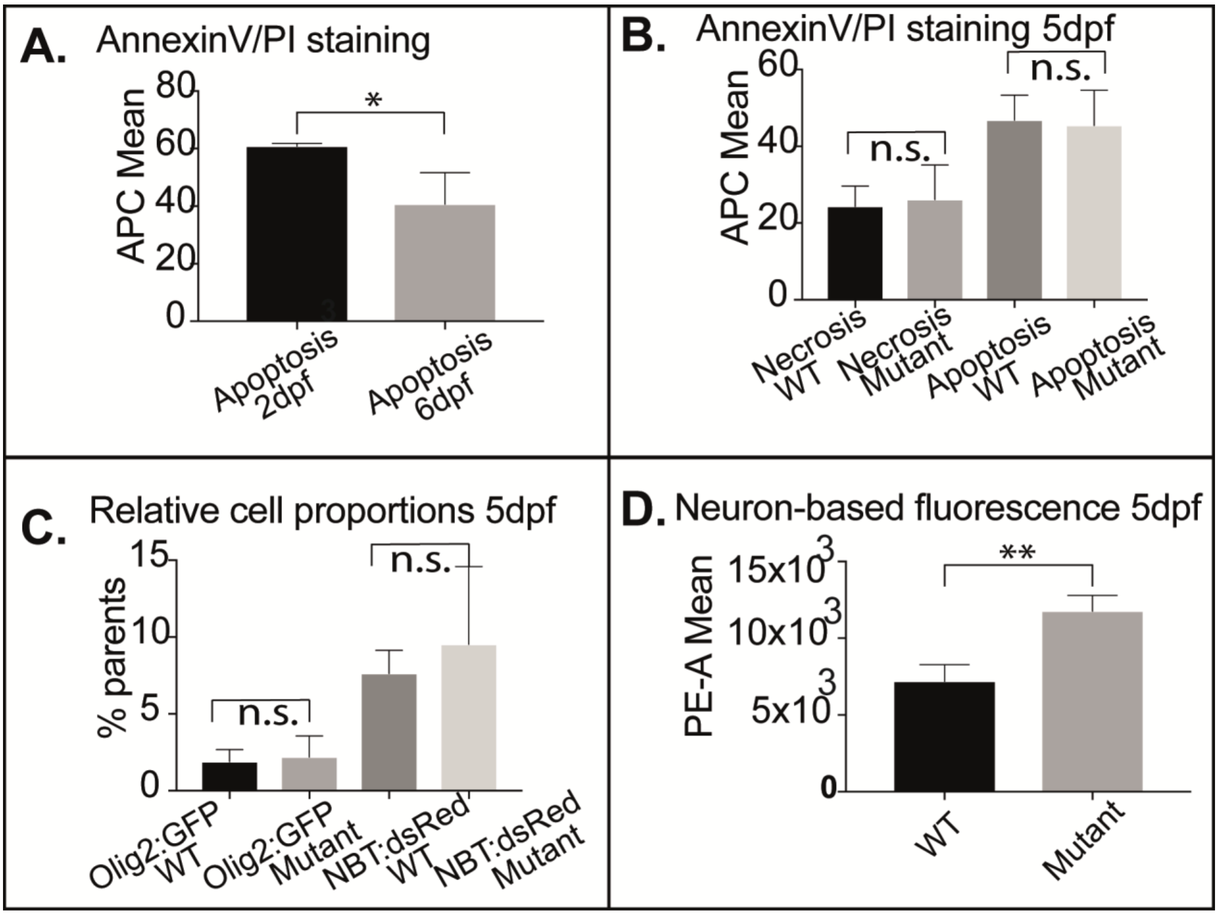

Figure S4

Cell death staining and quantification by flow cytometry in 5 dpf zebrafish larvae

Quantification of apoptosis and necrosis as determined by flow cytometry after dissociation of larvae and annexinV (aV)/propidium iodide (PI) cell staining. **A:** Quantification of cell death staining before (2dpf) and after (6dpf) developmental apoptosis in WT zebrafish. **B:** Quantification of cell death staining in WT and RNASET2-deficient larvae on 5 dpf revealed no differences (necrotic: WT 24,3%; mutants 26,1%; apoptotic: WT 46,8%; mutants 45,4%; n=19-20; p>0,5). **C:** Relative cell proportions of neurons and oligodendrocytes based on endogenous emission of fluorescence (Olig2:GFP & NBT:dsRed) without significant differences (neurons: WT 7,6%; SD 1,5; Mutants 9,5%; SD 5,0; p>0,5; n=3; OPCs: WT 1,9%; Mutants 2,2%; n=3; p>0,5). **D:** Increased fluorescence emitted from neurons (NBT:dsRed) in mutants (mean PE-A: WT 7192; Mutants 11766,7; n=3; p<0,01). N-numbers were generated from 10 litters and analyzed in 3 independent sessions.
